# Application of a person-centered prescription model improves pharmacotherapeutic indicators and reduces costs associated with pharmacological treatment in hospitalized older patients at the end of life

**DOI:** 10.3389/fpubh.2022.994819

**Published:** 2022-10-03

**Authors:** Alexander Ferro-Uriguen, Idoia Beobide-Telleria, Javier Gil-Goikouria, Petra Teresa Peña-Labour, Andrea Díaz-Vila, Arlovia Teresa Herasme-Grullón, Enrique Echevarría-Orella, Jesús Seco-Calvo

**Affiliations:** ^1^Department of Pharmacy, Ricardo Bermingham Hospital—Matia Foundation, Donostia-San Sebastian, Spain; ^2^Department of Physiology, University of the Basque Country (UPV/EHU), Bilbao, Spain; ^3^Network Centre for Biomedical Research in Mental Health to the Institute of Health Carlos III (CIBERSAM ISCIII), Madrid, Spain; ^4^Department of Geriatrics, Ricardo Bermingham Hospital—Matia Foundation, Donostia-San Sebastian, Spain; ^5^Institute of Biomedicine (IBIOMED), University of León, León, Spain; ^6^Department of Physiology, University of the Basque Country (UPV/EHU), Bilbao, Spain

**Keywords:** end of life (EOL), deprescribing, older people, palliative medicine, person-centered prescription

## Abstract

**Objective:**

This study sought to investigate whether applying an adapted person-centered prescription (PCP) model reduces the total regular medications in older people admitted in a subacute hospital at the end of life (EOL), improving pharmacotherapeutic indicators and reducing the expense associated with pharmacological treatment.

**Design:**

Randomized controlled trial. The trial was registered with ClinicalTrials.gov (NCT05454644).

**Setting:**

A subacute hospital in Basque Country, Spain.

**Subjects:**

Adults ≥65 years (*n* = 114) who were admitted to a geriatric convalescence unit and required palliative care.

**Intervention:**

The adapted PCP model consisted of a systematic four-step process conducted by geriatricians and clinical pharmacists. Relative to the original model, this adapted model entails a protocol for the tools and assessments to be conducted on people identified as being at the EOL.

**Measurements:**

After applying the adapted PCP model, the mean change in the number of regular drugs, STOPPFrail (Screening Tool of Older Persons' Prescriptions in Frail adults with limited life expectancy) criteria, drug burden index (DBI), drug–drug interactions, medication regimen complexity index (MRCI) and 28-days medication cost of chronic prescriptions between admission and discharge was analyzed. All patients were followed for 3 months after hospital discharge to measure the intervention's effectiveness over time on pharmacotherapeutic variables and the cost of chronic medical prescriptions.

**Results:**

The number of regular prescribed medications at baseline was 9.0 ± 3.2 in the intervention group and 8.2 ± 3.5 in the control group. The mean change in the number of regular prescriptions at discharge was −1.74 in the intervention group and −0.07 in the control group (mean difference = 1.67 ± 0.57; *p* = 0.007). Applying a PCP model reduced all measured criteria compared with pre-admission (*p* < 0.05). At discharge, the mean change in 28-days medication cost was significantly lower in the intervention group compared with the control group (−34.91€ vs. −0.36€; *p* < 0.004).

**Conclusion:**

Applying a PCP model improves pharmacotherapeutic indicators and reduces the costs associated with pharmacological treatment in hospitalized geriatric patients at the EOL, continuing for 3 months after hospital discharge. Future studies must investigate continuity in the transition between hospital care and primary care so that these new care models are offered transversally and not in isolation.

## Introduction

As the population ages, the number of people who will need palliative care is projected to increase notably in the coming decades due to multimorbidity and advanced chronicity. In the United Kingdom, a 14–25% increase in palliative care needs is expected by 2040, especially in patients with diseases such as dementia and cancer (1, 2). The complexity of care for older people, who have heterogeneous profiles and diverse needs, values, preferences, and therapeutic objectives, is increasing (3).

Alongside aging and chronicity, polypharmacy (defined as the use of ≥5 or ≥10 medications) is emerging as a major public health problem in older people, particularly those with advanced frailty and at the end-of-life (EOL). In a cross-sectional population study conducted in Spain between 2005 and 2015, people ≥80 years experienced the greatest increase in polypharmacy, from 11.7 to 36.7% (4). This is a cause for concern due to the observed association between polypharmacy and a range of negative health outcomes including drug-related problems, adverse drug events, physical and cognitive function, hospitalization, and mortality (5, 6). Increased polypharmacy is also expected to contribute to increased healthcare costs for both the patient and the healthcare system.

Although the scientific literature suggests new theoretical models for therapeutic approaches at the EOL (7, 8), the evidence indicates increased pharmacotherapy in the last year of life. A longitudinal cohort study conducted in Sweden reconstructed the drug prescription history of the last year of life in 511,843 older people (>65 years) (9) and found that the percentage exposed to ≥10 different medications increased from 30.3 to 47.2%. Polypharmacy increases in the last year of life, not only through additional medications to alleviate symptoms but also through additional long-term preventive treatments of questionable benefit (e.g., statins, calcium supplements, vitamin D, bisphosphonates, and antidementia drugs) (10).

The identification of older people with a limited life prognosis (11) is a key step in determining pharmacotherapeutic adequacy at the EOL and tailoring an individualized approach to each patient. For some researchers, a person-centered prescription model (PCP) (12–15) is the gold standard instrument for the care of people at the EOL, since it incorporates an individualized care plan based on the preferences and needs of the individual aimed at obtaining the main care objectives (prolongation of survival, maintenance of functionality or prioritizing symptom control) through shared decision-making. This model is based on a comprehensive geriatric assessment (CGA) (16) that allows the identification and quantification of physical, functional, psychological and social problems of the person and family to establish care goals and propose an individualized therapeutic plan. A recent systematic review summarizes strategies for optimization of pharmacotherapy at the EOL in three main categories (17): tools that describe a model or framework to approach deprescribing, tools that outline a deprescribing approach for the entire medication list, and tools that provide medication-specific advice. In a recent systematic review of the outcomes of deprescribing interventions at the EOL, satisfactory results were obtained in terms of medication appropriateness [defined as a reduction in unnecessary or potentially inappropriate prescriptions (PIPs)], but only two randomized clinical trial studies demonstrated improvement on this topic, making clear the need to continue investigating to generate a higher quality level of evidence (18).

In short, the application of this new model, rather than a strict application of clinical practice guidelines that are focused on each pathology, favors better symptomatic control of the disease and better quality of life. This requires the implementation of new strategies that approach a PCP model beyond care segregated by medical services, which, in current medicine, is inappropriate, uncoordinated and inefficient (19, 20).

This study's main objective was to investigate whether the application of an adapted PCP model during a hospital stay would reduce the total number of regular medications taken by older people at the EOL, improving pharmacotherapeutic indicators and reducing the expense associated with pharmacological treatment. We hypothesized that applying this modified method could optimize pharmacotherapeutic indicators and the expense associated with the pharmacological treatment of hospitalized patients.

## Methods

### Study design

This study was a parallel-group unblinded randomized clinical trial conducted in a subacute hospital in Gipuzkoa, Spain. Participants were randomized to receive either the usual pharmaceutical care or an adapted PCP model. The trial was registered with ClinicalTrials.gov (NCT05454644).

### Participants

All participants were aged ≥65 years and admitted to the geriatric convalescence unit of a subacute hospital, where they were identified according to their baseline in the first 24–72 h as having a non-oncological advanced chronic disease and being in need of palliative care, with a limited survival prognosis according to the necessity of palliative care (NECPAL) test (21). Patients with hospital stays of < 72 h, as well as those transferred to other hospitals or units and imminently terminal patients, were excluded.

### Randomization and data collection

Over 24 months (February 2018–February 2020), all patients with a positive NECPAL test who were admitted to the geriatric convalescence unit were selected consecutively and randomized to study groups at a 1:1 ratio. Randomization was stratified by geriatrician. The independent variables included and collected from the computerized clinical records of the Basque Health Service (Osakidetza) and the computerized records of the subacute hospital were: (i) sociodemographic characteristics, including gender, age, marital status, type of coexistence and Gijon socio-family assessment (22); (ii) clinical characteristics, including advanced chronic disease category, Charlson Comorbidity Index (23), Frail–VIG (24), cognitive assessment according to the Global Deterioration Scale–Functional Assessment Staging (GDS–FAST) (25), functional assessment according to the Barthel Index and the number of hospitalizations in the previous year and (iii) pharmacotherapeutic characteristics. Drug treatment data and variables related to pharmacotherapy were collected from primary care electronic prescriptions records of the Basque Health Service (Osakidetza) at hospital admission, discharge and during the study follow-up. Only regular prescriptions were recorded; those used on-demand or for a short time were recorded separately. Lastly, from the same source of drug treatment records and the December 2021 price list prescription (Nomenclator) of the Spanish Agency of Medicines and Health Products, the 28-day cost of prescriptions was estimated. For this, only the active prescription was taken into account at each time-point studied. Based on the retail price of the container of each prescribed medication, the unit price in € corrected by the patient's prescribed dose at each time-point was calculated. Subsequently, to find out 28-day cost of prescription, the corrected unit price was multiplied by 28.

### Intervention

**Figure 1:**
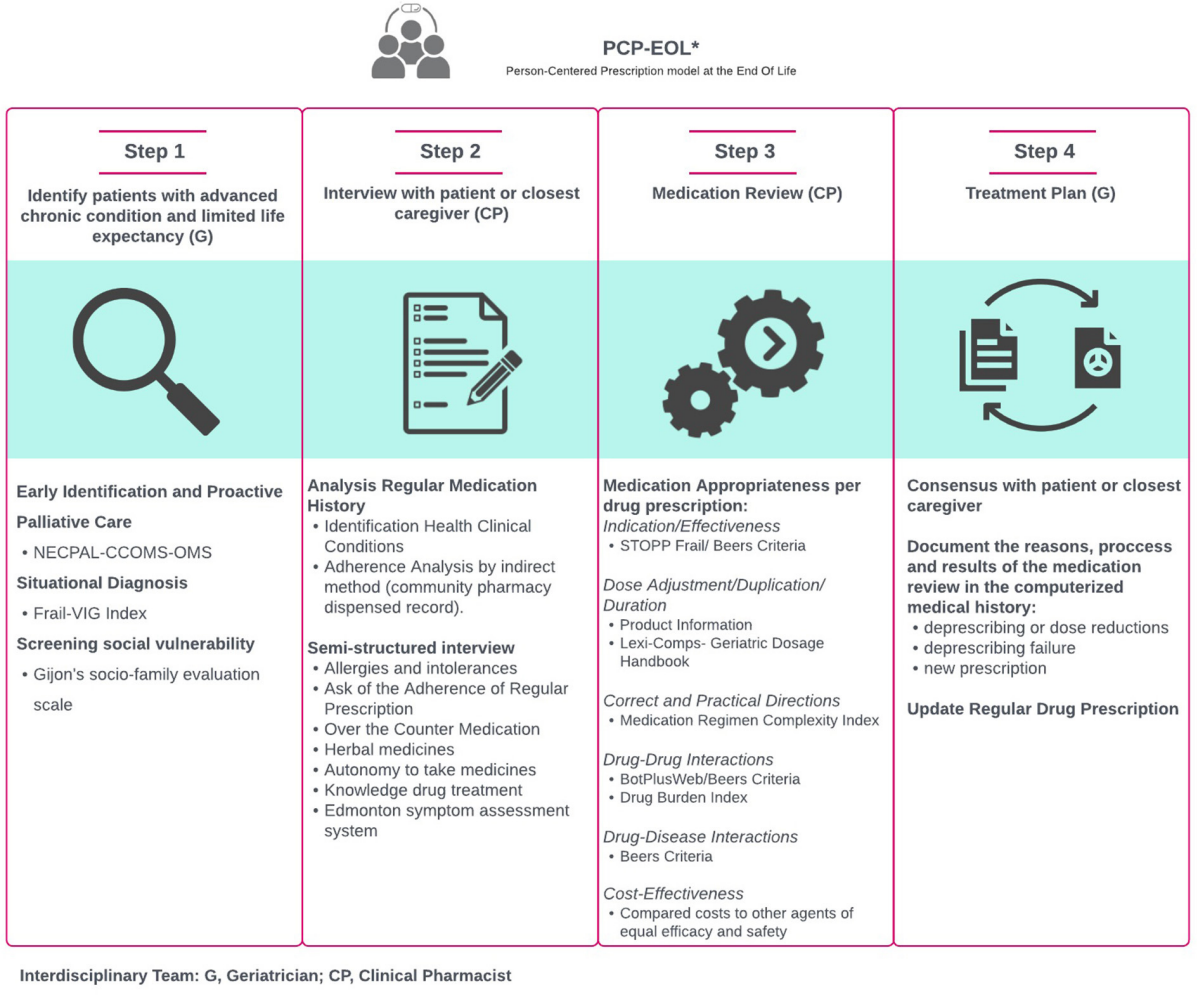
Person-centered prescription model at the end of life.

An interdisciplinary medicine-optimization strategy was implemented in people at the EOL based on the PCP model Espaulella-Panicot et al. (15) proposed. This is an adapted PCP model consisting of a systematic four-step process conducted by a geriatrician and a clinical pharmacist. Relative to the original model, this adapted model entails a protocol for the tools and assessments to be conducted on people identified as being at the EOL. The model is protocolized to gain external validity and possible future applicability in other hospitals or healthcare settings. It is described in detail in Figure 1.

#### Step 1

The geriatrician identified the patients as being at the EOL and determined the global-care goal for each patient after an initial comprehensive geriatric assessment performed within the first 24–72 h. Once the EOL situation is known, the clinical and care objectives are established (usually maintenance of functionality and/or symptom control and/or rarely the prolongation of survival) between health professionals and patients (or caregivers). Likewise, a screening for social vulnerability was conducted by collaborating with the social worker.

#### Step 2

Before interviewing each patient, the clinical pharmacist analyzed their pharmacotherapeutic history, electronic prescriptions from primary care and the last prescription sheet from their hospital of origin, if present. In addition, dispensations made by the community pharmacy in the previous 6 months were studied as an indirect measure of medication adherence.

The pharmacist performed a semi-structured interview to confirm the detailed up-to-date list of medications, directly assess treatment adherence, and analyze medication appropriateness. The patients or their main caregivers, if necessary, were asked about their autonomy in taking medication and their knowledge of the prescribed treatment. If the pharmacist detected any discrepancy with the medication prescribed at hospital admission, they reconciled the medication with the physician in charge. The pharmacist also asked about the control of the main symptoms according to the Edmonton symptom assessment system (26) to adapt the pharmacotherapy to the patient's situation.

#### Step 3

The clinical pharmacist conducted a structured medication review based on the medication appropriateness index (MAI) (27). The items studied by the MAI were analyzed with different indices and explicit criteria for each prescribed medication. These are indicated below:

- **Indication/effectiveness:** Product information, STOPPFrail (Screening Tool of Older Persons' Prescriptions in Frail adults with limited life expectancy) criteria (28) and Beers Criteria for Potentially Inappropriate Medication Use in Older Adults (29).
- **Dosage adjustment:** Product information and Lexi-Comp's *Geriatric Dosage Handbook*.
- **Correct and practical directions:** Medication Regimen Complexity Index (MRCI) (30), divided into three sections to analyze pharmaceutical forms, dose frequency, and additional instructions, which contribute to the final complexity score.
- **Drug–drug interactions:** Bot Plus (31)/Beers criteria and drug burden index (DBI) (32), which measures dose-dependent anticholinergic and sedative loads.
- **Drug–disease interactions:** Beers criteria.
- **Duplication, duration and cost-effectiveness:** Product information.

The pharmacist discussed any detected problems with the geriatrician until they reached a consensus and proposed a common pharmacotherapeutic approach.

#### Step 4

An individualized therapeutic plan was proposed to the patient and/or their closest caregiver. It is in this step when the patient (or main caregiver) expresses their preferences and needs based on their state of health and, together with the geriatrician, jointly deliberates on the decision regarding their pharmacological treatment. The reasons, process and results of the pharmacotherapeutic review (deprescribing or dose reductions, deprescribing failure or new prescriptions) were documented in the computerized medical record as well as the hospital discharge report to ensure the continuity of the intervention in the subsequent transition of care.

### Outcome measures

The primary outcome was the mean change in the number of regular medications between admission and discharge. *Pro re nata* (as-needed) medicines were not included. Combination products were included as one drug. Moreover, the mean changes between admission and discharge in STOPPFrail criteria, DBI, total drug–drug interactions and MRCI were measured. Any decrease in the pharmacotherapeutic variables studied during hospital admission was considered an optimization of the pharmacotherapy.

Although there are no objective measures, improvement in these pharmacotherapeutic indicators has been considered an indirect measure of quality of life (33, 34).

All patients were followed up with 1 month and 3 months after hospital discharge, and the same medication-related variables were recorded to measure the effectiveness of the PCP model over time, without performing any additional intervention on the subjects studied.

Likewise, the change in the 28-day cost of prescriptions in € was estimated between admission and discharge, as well as in the first 3 months after hospital discharge.

Other secondary outcomes were measured 3 months after discharge and included new emergency department presentations and unplanned hospital readmissions (see Supplementary Appendix 1).

### Sample size calculation and statistical analysis

The sample size estimation was based on the results of a study (35) and was carry out utilizing the G^*^Power-3.1.9.2 software (G^*^Power, Dusseldorf University, Germany). We calculated the statistical power of the trial to detect a mean difference of two regular medicines between the intervention and control groups (α = 0.05; 1-β = 0.8; SD = 3.8) at hospital discharge. Thus, the result recommended a minimum number of 46 subjects in each group (92 participants). Allowing for an estimated attrition rate (deaths and dropouts) of 25%, which is a characteristic in hospitalized geriatric patients at the EOL, we estimated that a sample size of 122 participants, with 61 in each group, would be required. We included only participants who completed the follow-up in the analysis of the primary outcome. Emergency department presentations and hospital admissions were determined on all randomized participants.

The selected variables were expressed as mean, median and frequency (percentages). Pearson's χ^2^ test was used to compare qualitative variables. Student's *t*-test and the Mann–Whitney *U*-test were used to compare parametric and non-parametric distributions, respectively.

During the follow-up phase, the effect of the passage of time was measured on the main pharmacotherapeutic variables using the repeated measures ANOVA test. We performed statistical analyses using SPSS software (SPSS Inc, Chicago, IL, version 20.0).

### Ethical considerations

The study (identify number: code AFU-PPG-2017-01) was approved by the Clinical Research Ethics Committee of the Gipuzkoa Health Area. Informed consent was previously obtained from all recruited patients. In cases where participants had cognitive impairment, consent was obtained from legal guardians who acted as surrogate informants.

## Results

**Figure 2:**
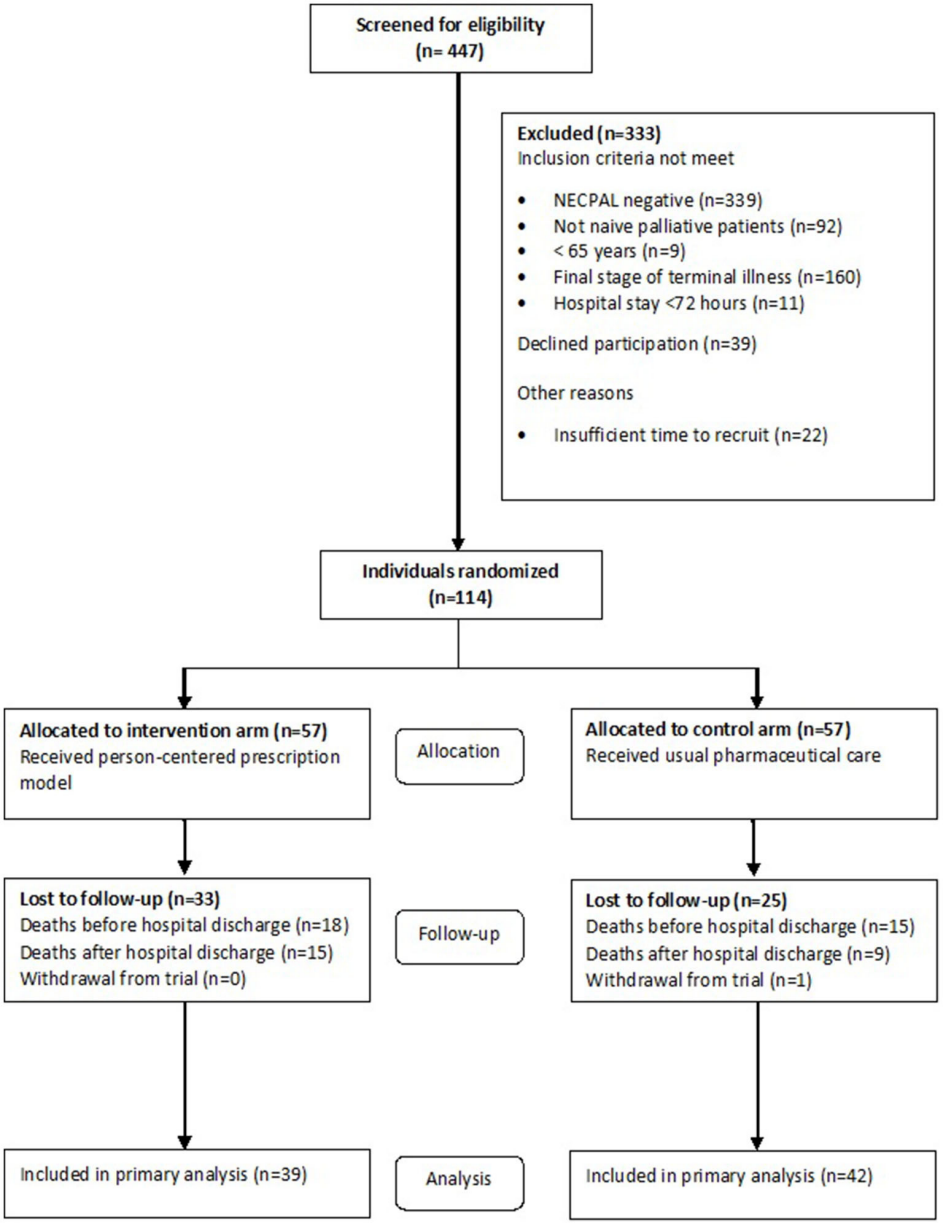
Recruitment and participation.

Figure 2 summarizes the flow of patients entered into the study and analyzed for primary and secondary outcomes. Study participants had a mean age of 87.7 (5.7) years, and 57.9% were female. No significant differences were present in the characteristics of the control (usual pharmaceutical care) and intervention (adapted PCP model) groups (Table 1). The hospital stay was 23.9 (14.4) for the control group and 27.0 (11.9) for the intervention group, with no significant differences (*p* = 0.225).

**Table 1:** Baseline characteristics of study participants.

**Variable**	**Control (*n* = 57)**	**Intervention (*n* = 57)**	***p*-Value**
Women, *n* (%)	30 (52.6)	36 (63.2)	0.255
Mean age, years (SD)	87.6 (5.7)	87.9 (5.7)	0.782
Marital status, *n* (%)			0.363
- Unmarried, divorced, separated	7 (12.3)	7 (12.3)	
- Married	17 (29.8)	24 (42.1)	
- Widowed	33 (57.9)	26 (45.6)	
Type of co-existence, *n* (%)			0.568
- Alone	12 (21.2)	11 (19.3)	
- Spouse	17 (29.8)	24 (42.1)	
- Children or other relatives	20 (35.1)	15 (26.3)	
- Other caregivers	8 (14.0)	7 (12.3)	
Gijón's socio-family assessment, *n* (%)			0.235
- Good social status	10 (17.5)	5 (8.8)	
- Social risk	34 (59.6)	42 (73.7)	
- Social problem	13 (22.8)	10 (17.5)	
Place of provenance, *n* (%)			0.508
- Hospital	51 (89.5)	53 (93.0)	
- Primary care/Nursing home	6 (10.5)	4 (7.0)	
Illness trajectories, *n* (%)			0.700
- Dementia, frailty, neurological disease	37 (64.9)	34 (59.6)	
- Organic failure	16 (28.1)	20 (35.1)	
- Combined	4 (7.0)	3 (5.3)	
CCI, median (IQR)	4.0 (3.0)	4.0 (3.0)	0.527
No. of patients with ≥3 points CCI, *n* (%)	43 (75.4)	42 (73.7)	0.830
Diagnoses, *n* (%)			
- Myocardial infarction	9 (15.8)	8 (14.0)	0.793
- Congestive heart failure	26 (45.6)	30 (52.6)	0.454
- Peripheral vascular disease	4 (7.0)	10 (17.5)	0.087
- Cerebrovascular accident	19 (33.3)	15 (26.3)	0.413
- Dementia	30 (52.6)	31 (54.4)	0.851
- Chronic obstructive pulmonary disease	14 (24.6)	14 (24.6)	1.000
- Diabetes mellitus	23 (40.4)	20 (35.1)	0.562
- Moderate to severe chronic kidney disease	27 (47.4)	28 (49.1)	0.851
- Cancer without metastases	7 (12.3)	8 (14.0)	0.782
GDS ≥6, *n* (%)	21 (36.8)	20 (35.1)	0.845
Barthel index, median (IQR)	40 (36)	34 (63)	0.664
Barthel index ≤ 35, *n* (%)	24 (42.1)	30 (52.6)	0.260
Frail-VIG, mean (SD)	0.50 (0.15)	0.52 (0.12)	0.368
Frail-VIG >0.50, *n* (%)	27 (47.4)	34 (59.6)	0.189
Geriatric syndromes, *n* (%)			
- Insomnia/anxiety^a^	40 (70.2)	41 (71.9)	0.836
- Delirium^b^	10 (17.5)	18 (31.6)	0.082
- Falls^c^	17 (29.8)	22 (28.6)	0.324
- Pressure ulcers	10 (17.5)	16 (28.1)	0.180
- Disphagia	22 (38.6)	25 (43.9)	0.568
- Malnutrition (≥5% weight loss in the last 6 months)	14 (24.6)	17 (29.6)	0.528
Hospitalization in the last year, *n* (%)			0.621
- 0	23 (40.4)	23 (40.4)	
- 1	21 (36.8)	17 (29.8)	
- ≥2	13 (22.8)	17 (29.8)	
Mean (SD) number of days between admission and hospital discharge	23.9 (14.4)	27.0 (11.9)	0.225
Medication use			
- No. of regular medications, mean (SD)	8.2 (3.5)	9.0 (3.2)	0.158
- No. of patients with ≥10 regular medications, *n* (%)	16 (28.1)	24 (42.1)	0.116
STOPP Frail-defined PIMs, mean (SD)	1.7 (1.4)	2.0 (1.4)	0.322
No. of patients with ≥2 STOPPFrail-defined PIMs, *n* (%)	27 (47.4)	35 (61.4)	0.132
DBI, mean (SD)	0.99 (0.82)	1.22 (0.83)	0.143
No. of patients with ≥1 point DBI, *n* (%)	27 (47.4)	35 (61.4)	0.132
Total drug-drug interactions			
- Severe drug-drug interactions, mean (SD)	3.72 (3.85)	4.35 (3.39)	0.354
- Moderate drug-drug interactions, mean (SD)	1.88 (2.65)	2.53 (2.44)	0.176
- Mild drug-drug interactions, mean (SD)	1.60 (1.73)	1.56 (1.43)	0.906
MRCI, mean (SD)	28.1 (13.7)	31.5 (11.5)	0.158
Autonomy to take medication, *n* (%)			0.825
- Self-management	5 (9.1)	4 (7.0)	
- Supervision	13 (23.6)	16 (28.1)	
- Dependent	37 (67.3)	37 (64.9)	

CCI, Charlson comorbidity index; DBI, drug burden index; Frail-VIG, frailty index based on comprehensive geriatric assessment; GDS, Reisberg's global deterioration scale; IQR, interquartile range; MRCI, medication regimen complexity index; PIM, potentially inappropriate medications; SD, standard deviation.

^a^Need benzodiazepines or other psychotropics profile sedative for insomnia/anxiety. ^b^Delirium or behavioral disorder that has required taking neuroleptics in the last 6 months. ^c^≥2 falls or a fall requiring hospitalization in the last 6 months.

Intervention group patients (*n* = 39) and control group patients (*n* = 42) received a mean of 8.46 (±3.27), and 8.05 (±3.13) regular prescription medications, respectively, at baseline. The mean change in the number of regular prescriptions at discharge was significant at −1.74 (±2.75) in the intervention group and −0.07 (±2.37) in the control group (mean difference = 1.67 (±0.57); 95% confidence interval (CI) = 0.54–2.81; *p* < 0.007) (Table 2).

**Table 2:** Effectiveness in the pharmacotherapeutics variables after the application of the person-centered prescription model.

**Variable**	**Control** **(*****n*** = **42)**^**a**^			**Intervention** **(*****n*** = **39)**^**a**^			**Mean difference. Change between groups**	**CI**	***p*-Value**
	**Admission**	**Discharge**	**Difference**	**Admission**	**Discharge**	**Difference**			
No. of regular medications, mean (SD)	8.05 (3.13)	7.98 (3.46)	−0.07 (2.37)	8.46 (3.27)	6.72 (2.76)	−1.74 (2.75)	1.67 (0.57)	0.54–2.81	0.007*
STOPP Frail-defined PIMs, mean (SD)	1.74 (1.38)	1.29 (1.31)	−0.45 (0.80)	1.69 (1.36)	0.15 (0.37)	−1.54 (1.29)	1.09 (0.24)	0.60–1.57	< 0.001*
DBI, mean (SD)	1.00 (0.70)	1.02 (0.64)	0.02 (0.42)	1.27 (0.82)	1.01 (0.71)	−0.26 (0.63)	0.28 (0.12)	−0.04–0.52	0.010*
Total drug-drug interactions, mean (SD)	3.24 (3.07)	3.31 (3.58)	0.70 (3.60)	3.79 (3.25)	2.28 (2.67)	−1.51 (2.15)	1.58 (0.66)	0.26–2.90	< 0.001*
MRCI, mean (SD)	27.18 (12.70)	28.71 (13.17)	1.53 (9.02)	29.28 (11.33)	23.68 (10.02)	−5.60 (9.18)	7.14 (2.02)	3.11–11.17	0.001*

CI, confidence interval; DBI, drug burden index; MRCI, medication regimen complexity index; PIM, potentially inappropriate medications; SD, standard deviation.

^a^Only participants who were alive at hospital discharge were included.

*p < 0.05.

At the same time, the pharmacotherapeutics variables of the STOPPFrail criteria (*p* < 0.01), DBI (*p* = 0.01), total drug–drug interactions (*p* < 0.01), and MRCI (*p* < 0.01) improved significantly between admission and discharge in the intervention group compared to the control group (Table 2).

In patients alive 3 months after hospital discharge, the effect of the passage of time was measured on the different pharmacotherapeutic variables. The STOPPFrail criteria and drug–drug interactions remained stable over time for both the control and intervention groups (Table 3). However, after hospital discharge and with time, the number of chronic medications, DBI and MRCI increased significantly again in the control group and the intervention group.

**Table 3:** Effect of time in pharmacotherapeutic variables after the application of the person-centered prescription.

**Variable**	**Control** **(*****n*** = **32)**^**a**^			**Intervention** **(*****n*** = **24)**^**a**^			**Effect of time *p*-Value^†^**	**Effect of time and groups *p-*Value^†^**
	**Discharge**	**1st month**	**3rd month**	**Discharge**	**1st month**	**3rd month**		
No. of regular medications, mean (SD)	7.73 (3.36)	7.45 (3.55)	8.15 (3.51)	6.54 (2.70)	7.04 (2.64)	7.33 (2.50)	0.005*	0.187
STOPP Frail-defined PIMs, mean (SD)	1.24 (1.17)	1.24 (1.17)	1.18 (1.13)	0.08 (0.28)	0.08 (0.28)	0.21 (0.51)	0.370	0.935
DBI, mean (SD)	1.06 (0.65)	1.10 (0.67)	1.11 (0.67)	1.09 (0.78)	1.23 (0.82)	1.35 (0.78)	0.005*	0.128
Total drug-drug interactions, mean (SD)	3.53 (4.00)	3.67 (3.92)	3.63 (4.06)	2.04 (1.99)	1.96 (1.92)	2.22 (2.00)	0.533	0.117
MRCI, mean (SD)	27.20 (12.65)	27.53 (12.35)	28.53 (12.08)	22.17 (9.67)	23.73 (9.16)	25.94 (9.18)	0.001*	0.136

DBI, drug burden index; MRCI, medication regimen complexity index; PIM, potentially inappropriate medications; SD, standard deviation.

^a^Only participants who were alive at 3rd month after hospital discharge were included.

^†^Wilks's Lambda; *p < 0.05.

At baseline, there were no statistically significant differences in the extrapolated mean monthly medication costs between the control and intervention groups, (97.87€ ± 55.28 and 133.43€ ± 70.15, respectively; *p* = 0.269). At discharge, the mean change in monthly medication cost was significantly lower in the intervention group (−34.91€ ± 60.21) compared with the control group (−0.36€ ± 36.94) (mean difference = 34.55€ ± 11.42; 95% CI = 11.75–57.36; *p* < 0.004). This decrease continued for 3 months after discharge, and no changes were observed in the 28-day prescription cost over time (*p* = 0.569), nor over time between groups (*p* = 0.424).

## Discussion

Our results show that the application of a PCP model in people identified as being at the EOL during their stay in a subacute hospital significantly reduced the consumption of chronic medications and their complexity, drug–drug interactions, the number of STOPPFrail criteria and anticholinergic and sedative loads compared with before admission. Likewise, this intervention significantly reduced the cost of patients' chronic medical prescriptions.

The application of PCP models is clearly established (37–41) in the scientific literature and can identify inappropriate prescriptions, optimize polypharmacy and improve medication adherence in different profiles of older patients. Additionally, the use of tools with explicit criteria [OncPal (42) and STOPPFrail (28)] to determine inappropriate drugs at the EOL is valid for deprescription and produces beneficial effects (43). We wanted to adapt the PCP model to the cases of people identified as being at the EOL with advanced non-oncological chronic conditions and to migrate from a theoretical to a practical model, proposing the use of concrete assessments and specific tools and protocolling their use.

In addition, the effect of the model's application remained stable during the first 3 months after hospital discharge, mainly for the STOPPFrail criteria and generally related to drugs that the patient persistently failed to take or tolerate (criterion A1), drugs with no clear valid clinical indication (criterion A2) and other explicit criteria for preventive drugs used with an unfavorable benefit balance at the EOL. The number of medications prescribed is the most important predictor of iatrogenic harm (44); therefore, any efforts to reduce PIP may improve patients' clinical status. Hitherto, the different deprescription tools published for EOL patients describe potential for mortality reduction and cost savings (18).

Regarding the amount of regular drug consumption, the effect of our intervention was slightly lower than that found in a recently published study (45) (an average decrease of 1.7 vs 2.6 medications) during hospital admission. In that study, the researchers started with a more polymedicated population and, presumably, accumulated a greater number of PIPs that could be withdrawn. However, our study allowed us to verify how prescriptions, as well as the intake of chronic medications, have been increasing over time in this type of patient after discharge. This phenomenon may be linked to an increase in drugs used for symptomatic control of the disease because, as we have previously mentioned, the STOPPFrail criteria, which are mainly related to the use of preventive drugs, remained stable for 3 months after hospital discharge.

Drug–drug interactions are frequently associated with adverse drug reactions. On multiple occasions, they are the reason for new hospitalizations or visits to the emergency room (36, 46–48). However, in our study, the total number of drug–drug interactions detected also remained stable 3 months after applying the PCP model, so the reduction observed during hospital admission is considered a robust result in terms of improving the quality of prescription and possible improvement in the clinical and health status of intervention group patients.

Furthermore, the majority of older people are exposed to drugs with anticholinergic and/or sedative effects (49), and resorting to anticholinergics and sedatives to control pain, dyspnea, secretions and patient anxiety, among other symptoms, is common in palliative care (50). Thus, in our study, most of the anticholinergic and/or sedative drugs prescribed at the EOL are related to drugs for symptomatic use, and their use increased as the patient approached the EOL, which is consistent with previous studies (51, 52). This could be due to the increase of DBI during patient follow-up, which could be related to an increase in psychotropic medications for symptom management at EOL. However, greater exposure to drugs with anticholinergic activity was associated with more fatigue, dry mouth, worse concentration and worsening status at the EOL (52). Further research in this line is needed to clarify whether anticholinergic load directly causes this worsening status or that people who are worsening need more medication to optimize their state of comfort.

Finally, regarding the pharmaceutical expense of caring for these patients, our results show an average reduction of 19% in drug costs over 28 days, a figure slightly lower than that recorded by Curtin et al. (45), who found a 28% reduction for the same measurement variable. In our study, the intervention was maintained for at least 3 months; therefore, given the exponential increase in people with advanced chronic diseases, these new models are relevant to optimize economic resources, thus avoiding futile and inappropriate therapies.

Our study has the following limitations. First, only patients in a single subacute hospital were recruited, which makes the results difficult to generalize to the broader population. Second, the physicians simultaneously received patients from the intervention group and the control group throughout the study period, so the patients assigned to the control group could have experienced the effects of the intervention due to a training effect. Third, the trial is underpowered to detect differences in health-related outcomes (emergency department presentation and unplanned hospital admission) between the intervention and control groups. Furthermore, with the outbreak of the COVID19 pandemic and the exceptional prevention and protection measures adopted at the hospital in March 2020, the 122 patients proposed at the start of the study could not be recruited. Thus, the study stopped with 114 patients recruited, a very close number to that estimated in the sample size calculation.

However, this work also had notable strengths. The effectiveness of the proposed model's intervention generates a greater body of evidence for people at the EOL who are systematically excluded from other research. We must also highlight the context of medication appropriateness in patients admitted to a subacute hospital, in which evidence is scarce. As a point in favor, we must consider that a prolonged hospital stay, as in the studied cases, allows medication appropriateness in a controlled environment, where medications are frequently deprescribed and the effects of withdrawal can be monitored. In short, working within an interdisciplinary team of physicians, nurses, pharmacists, social workers and other health professionals allows us to provide an effective, safe and high-quality response adapted to the real clinical and personal situations of each patient.

In conclusion, the application of a PCP model reduces pharmacotherapeutic indicators, improves the quality of life and reduces costs associated with pharmacological treatment in hospitalized geriatric patients at the EOL. Our results showed the effectiveness of the adequacy of medication in people identified in this vital situation, maintaining its effect in the first 3 months after hospital discharge. It has also meant an optimization of economic resources with a notable decrease in pharmaceutical spending. In future studies, it will be necessary to guarantee the continuity of the care transitions between hospital and primary care, so that these new care models are offered transversally and not in isolation.

## Data availability statement

The raw data supporting the conclusions of this article will be made available by the authors, without undue reservation.

## Ethics statement

The study (Identify number: Code AFU-PPG-2017-01) was approved by the Clinical Research Ethics Committee of the Gipuzkoa Health Area. The patients/participants provided their written informed consent to participate in this study.

## Author contributions

AF-U, IB-T, JG-G, and EE-O formulated the research question and contributed to the conception and design of the work. AF-U, PP-L, AD-V, and AH-G applied the intervention of the person-centered prescription model and facilitated data collection. AF-U, IB-T, JG-G, EE-Q, and JS-C analyzed the data and critically reviewed the manuscript. The first draft of the manuscript was written by AF-U. All authors contributed to the article and approved the submitted version.

## Conflict of interest

The authors declare that the research was conducted in the absence of any commercial or financial relationships that could be construed as a potential conflict of interest.

## Publisher's note

All claims expressed in this article are solely those of the authors and do not necessarily represent those of their affiliated organizations, or those of the publisher, the editors and the reviewers. Any product that may be evaluated in this article, or claim that may be made by its manufacturer, is not guaranteed or endorsed by the publisher.
