# Supplementary material for: Application of a person-centered prescription model improves pharmacotherapeutic indicators and reduces costs associated with pharmacological treatment in hospitalized older patients at the end of life
Source: Front Public Health. 2022 Oct 3;10:994819. doi: 10.3389/fpubh.2022.994819 (PMC9574095; doi:10.3389/fpubh.2022.994819)
Supplement: Supplementary file 1 [file Table_1.DOCX]

SupplementaryTable. Effect of application of person-centred prescription model on secondary outcomes three months after hospital discharge.

| Outcome (ITT) | Control  (n= 57) | | Intervention (n= 57) | |  |  |
| --- | --- | --- | --- | --- | --- | --- |
|  | Proportion (95% CI) | Number of participants | Proportion (95% CI) | Number of participants | Relative Risk (95%) | p |
| ED presentation | 0.16 (0.06-0.26) | 9 | 0.16 (0.06-0.26) | 9 | 1.00 (0.43-2.33) | 1.000 |
| Unplanned hospital admission | 0.23 (0.12-0.34) | 13 | 0.19 (0.09-0.30) | 11 | 0.85 (0.41-1.73) | 0.646 |

ITT, intention to treat analysis; ED, Emergency Department; SD, standard deviation; CI, Confidence Interval
*p<0.05
